# Comparative Effectiveness of Post-extubation Respiratory Support Strategies in Preventing Reintubation and Respiratory Failure in Critically Ill Adults: A Systematic Review and Network Meta-Analysis

**DOI:** 10.7759/cureus.112264

**Published:** 2026-07-08

**Authors:** Amr K Abdeltawab, Hind A Tolba, Falah F Alsahli, Mansour A Albalawi, Abdulaziz K Altowairqi, Esraa M Abdelrahman, Alwaleed H Almawash, Ali S Metwaly

**Affiliations:** 1 Intensive Care Unit, Al-Thaghr Hospital, Jeddah, SAU; 2 Respiratory Medicine, Batterjee Medical College, Jeddah, SAU; 3 Medicine, Al-Jouf University, Sakaka, SAU; 4 General Practice, University of Hail, Hail, SAU; 5 Critical Care, Alhada Armed Forces Hospital, Taif, SAU; 6 Medicine, Kulhudhuffushi Regional Hospital, Kulhudhuffushi, MDV; 7 Medicine, Arabian Gulf University, Riyadh, SAU; 8 Precision Medicine, Faculty of Pharmacy, Alexandria University, Alexandria, EGY

**Keywords:** critical care, extubation failure, high-flow nasal cannula, mechanical ventilation, network meta-analysis, noninvasive ventilation, reintubation

## Abstract

Post-extubation respiratory failure requiring reintubation is associated with substantial morbidity and mortality in critically ill patients. The comparative effectiveness of available non-invasive respiratory support strategies, conventional oxygen therapy (COT), high-flow nasal cannula (HFNC), non-invasive ventilation (NIV), and the combination of NIV and HFNC (NIV+HFNC), remains debated. This systematic review and network meta-analysis (NMA) aimed to determine the comparative effectiveness of post-extubation respiratory support modalities in preventing reintubation in critically ill adults. MEDLINE, Embase, the Cochrane Central Register of Controlled Trials (CENTRAL), the Cumulative Index to Nursing and Allied Health Literature (CINAHL), and Scopus were searched from 1 January 2010 to 31 May 2026. Randomized controlled trials (RCTs) and adjusted non-randomized comparative studies evaluating post-extubation respiratory support applied within 24 hours of planned extubation in adult intensive care unit (ICU) patients were eligible for inclusion. Twenty-eight studies (22 RCTs and six observational studies) met the inclusion criteria. The NMA demonstrated that all active respiratory support strategies significantly reduced the risk of reintubation compared to COT. The combined NIV+HFNC strategy was ranked as the most effective intervention (P-score=0.81; risk ratio (RR) vs. COT: 0.53 (95% CI: 0.33-0.84); low certainty), followed closely by NIV alone (P-score=0.79; RR vs. COT: 0.55 (95% CI: 0.41-0.76); low certainty) and HFNC alone (P-score=0.40; RR vs. COT: 0.68 (95% CI: 0.52-0.89); moderate certainty). There were no statistically significant differences in reintubation rates between the active interventions (NIV+HFNC vs. HFNC: RR 0.78 (0.50-1.22); NIV vs. HFNC: RR 0.81 (0.63-1.05)). In critically ill adults undergoing planned extubation, active non-invasive respiratory support (HFNC, NIV, or their combination) reduces the risk of reintubation compared to COT. The sequential combination of NIV and HFNC ranked highest probabilistically, though direct comparisons between active interventions lacked statistical significance. The choice of support should be individualized according to patient risk profile, clinical context, tolerance, and certainty of available evidence.

## Introduction and background

Discontinuing invasive mechanical ventilation is a critical transition in the intensive care unit (ICU) [1]. Despite the successful completion of spontaneous breathing trials, extubation failure, defined as the need for reintubation within 48-72 hours, remains a common complication, occurring in 10-20% of general ICU patients and up to 30-48% of high-risk populations [2,3]. Extubation failure is an independent risk factor for devastating clinical consequences, including ventilator-associated pneumonia, prolonged ICU and hospital lengths of stay (LOS), elevated healthcare costs, and an increased mortality rate that can approach 50% [3-5]. Optimizing post-extubation respiratory support to prevent reintubation is a clinical priority.

Prophylactic respiratory support following extubation relies on conventional oxygen therapy (COT). However, COT fails to meet the peak inspiratory flow demands of critically ill patients and delivers cold, dry gas that can impair mucociliary clearance and induce patient discomfort [1,6]. Non-invasive ventilation (NIV) has been advocated to bridge this gap, particularly in high-risk patients, as it provides positive end-expiratory pressure (PEEP) to prevent alveolar collapse and pressure support to unload respiratory muscles [7]. Despite these physiological benefits, the clinical efficacy of NIV is often hampered by poor patient tolerance, claustrophobia, facial skin breakdown, and gastric distension [6,8]. In recent years, high-flow nasal cannula (HFNC) has emerged as an attractive and less restrictive alternative. HFNC delivers heated and humidified oxygen at high flow rates (up to 60 L/min), matches patient inspiratory demands, washes out the nasopharyngeal dead space, and generates a flow-dependent PEEP [9]. HFNC is associated with superior patient comfort and fewer interface-related adverse events than NIV [8,10].

Despite the theoretical advantages of these modalities, the optimal post-extubation respiratory support strategy remains highly debated [11,12]. Current clinical practice guidelines conditionally recommend NIV for high-risk patients, particularly those with hypercapnia or chronic obstructive pulmonary disease (COPD), and suggest HFNC for low-risk or post-surgical patients [2,13]. However, recent pairwise meta-analyses and large randomized controlled trials (RCTs) have yielded conflicting results regarding the comparative superiority of HFNC versus NIV [4,10]. Some systematic reviews conclude that HFNC is non-inferior to NIV for preventing reintubation and is better tolerated [13,14], whereas others suggest that NIV, or a sequential combination of NIV and HFNC, provides superior protection against post-extubation respiratory failure [3,15].

These discordant findings highlight a knowledge gap that traditional pairwise meta-analyses cannot fully resolve. Network meta-analysis (NMA) allows for the simultaneous evaluation of three or more interventions by synthesizing both direct (head-to-head) and indirect evidence, thereby providing a robust hierarchy of treatment efficacy [11,13]. Therefore, a systematic review and NMA of RCTs and eligible comparative non-randomized studies were conducted to evaluate the comparative effectiveness and safety of post-extubation respiratory support strategies, including COT, HFNC, NIV, and their combination, in preventing reintubation and post-extubation respiratory failure in critically ill adults.

## Review

Methods

Protocol and Registration

This systematic review and NMA was conducted in adherence to the Preferred Reporting Items for Systematic Reviews and Meta-Analyses (PRISMA) 2020 guidelines [16] and the PRISMA extension for Network Meta-Analyses (PRISMA-NMA) [17]. The study protocol was prospectively registered with the International Prospective Register of Systematic Reviews (PROSPERO) (registration number: CRD420261326702).

Search Strategy

A systematic literature search was developed in consultation with a medical information specialist. Five major electronic databases were searched: MEDLINE (via PubMed), Embase, the Cochrane Central Register of Controlled Trials (CENTRAL), the Cumulative Index to Nursing and Allied Health Literature (CINAHL), and Scopus. To capture contemporary practice patterns corresponding with the widespread adoption of high-flow oxygen technologies, the search was restricted to articles published between 1 January 2010 and 31 May 2026. The search syntax incorporated Medical Subject Headings (MeSH), Emtree terms, and natural language keywords mapped to the PICO framework, including combinations of "non-invasive ventilation", "high-flow nasal cannula", "continuous positive airway pressure", "extubation", and "reintubation". Backward citation tracking of included studies and relevant review articles was also performed to identify additional eligible studies.

Eligibility Criteria

Study inclusion was governed by the predefined PICO criteria. The population was critically ill adult patients (≥18 years of age) requiring invasive mechanical ventilation for acute respiratory failure for a minimum of 24 hours, who underwent planned extubation following the successful completion of a spontaneous breathing trial (SBT).

The intervention was post-extubation respiratory support applied within 24 hours of extubation, comparing at least two of the following modalities: NIV, continuous positive airway pressure (CPAP), HFNC, and COT (e.g., nasal cannula or Venturi mask). We evaluated these interventions, which were applied either prophylactically or as rescue therapy.

The primary outcome was the incidence of reintubation within 48-72 hours post-extubation, while the secondary outcomes included the incidence of post-extubation respiratory failure, ICU and hospital LOS, ventilator-free days, ICU and hospital mortality (at 28 and 90 days), and adverse events or patient comfort.

RCTs (parallel or cluster-crossover) and non-randomized comparative studies (e.g., prospective or retrospective cohort studies with contemporaneous control groups or propensity score matching) were included, while single-arm studies and pediatric trials were excluded.

Study Selection and Data Extraction

Two independent reviewers screened all retrieved citations based on their titles and abstracts, followed by a full-text review of potentially eligible articles. Inter-rater reliability (IRR) for study inclusion was quantified using Cohen's kappa coefficient (κ). Data extraction was performed independently and in duplicate using a standardized electronic form. The extracted data included study characteristics, patient demographics, baseline severity scores (Acute Physiology and Chronic Health Evaluation (APACHE) II and Sequential Organ Failure Assessment (SOFA)), extubation parameters, and relevant clinical outcomes. Discrepancies at any stage were resolved through adjudication by a third senior methodologist.

Risk of Bias Assessment

The methodological quality of the included studies was independently appraised by two investigators. RCTs were evaluated using the revised Cochrane Risk of Bias 2 (RoB 2) tool, assessing domains such as the randomization process, deviations from intended interventions, missing outcome data, measurement of the outcome, and selection of the reported result [18]. Non-randomized comparative studies were evaluated using the Risk of Bias in Non-randomized Studies of Interventions (ROBINS-I) tool [19].

Statistical Analysis and NMA

All statistical analyses were performed using R (Version 4.6.0; R Foundation for Statistical Computing, Vienna, Austria) [20] with the meta and netmeta packages [21]. For direct pairwise comparisons, the data were pooled using a DerSimonian-Laird random-effects model [22]. Effect sizes for dichotomous outcomes (e.g., reintubation, mortality) were expressed as risk ratios (RR) with 95% confidence intervals (CIs). Continuous outcomes (e.g., LOS) were analyzed using mean differences (MD) with 95% CIs.

A frequentist random-effects NMA was conducted to simultaneously compare all respiratory support modalities. The network geometry was visually represented using network plots, wherein the node sizes were proportional to the number of patients and the edge thickness reflected the number of direct comparisons. The relative rankings of the treatment modalities were estimated using the surface under the cumulative ranking curve (SUCRA) and rankograms, where a SUCRA value approaching 100% indicates that an intervention is virtually certain to be the most effective [23].

Heterogeneity, Inconsistency, and Publication Bias

Statistical heterogeneity across the network was quantified using Cochran's Q test, the I^2^ statistic, and τ^2^ (tau-squared). An I^2^ value of >50% was indicative of substantial heterogeneity. The fundamental transitivity assumption of the NMA was assessed by comparing the distribution of potential clinical effect modifiers (e.g., baseline APACHE II scores, proportion of COPD patients) across the available direct comparisons. To assess the inconsistency between direct and indirect evidence, we employed the node-splitting method for local inconsistency [24] and a design-by-treatment interaction model for global inconsistency.

To explore potential sources of heterogeneity, random-effects meta-regression (for continuous variables such as age and intubation duration) and subgroup analyses (e.g., medical vs. surgical ICU, prophylactic vs. rescue application, and high-risk vs. low-risk) were performed. A leave-one-out sensitivity analysis was conducted to assess the robustness of pooled estimates. Small-study effects and publication bias were evaluated using comparison-adjusted funnel plots and Egger's rank correlation test when 10 or more studies were available for a specific outcome [25].

Certainty of Evidence

The certainty of the evidence for primary and critical secondary outcomes was evaluated using the Confidence in Network Meta-Analysis (CINeMA) framework, an adaptation of the Grading of Recommendations Assessment, Development, and Evaluation (GRADE) approach designed specifically for NMA [26]. The evidence was downgraded based on within-study bias, reporting bias, indirectness, imprecision, heterogeneity, and network incoherence.

Results

Study Selection and IRR

**Figure 1:**
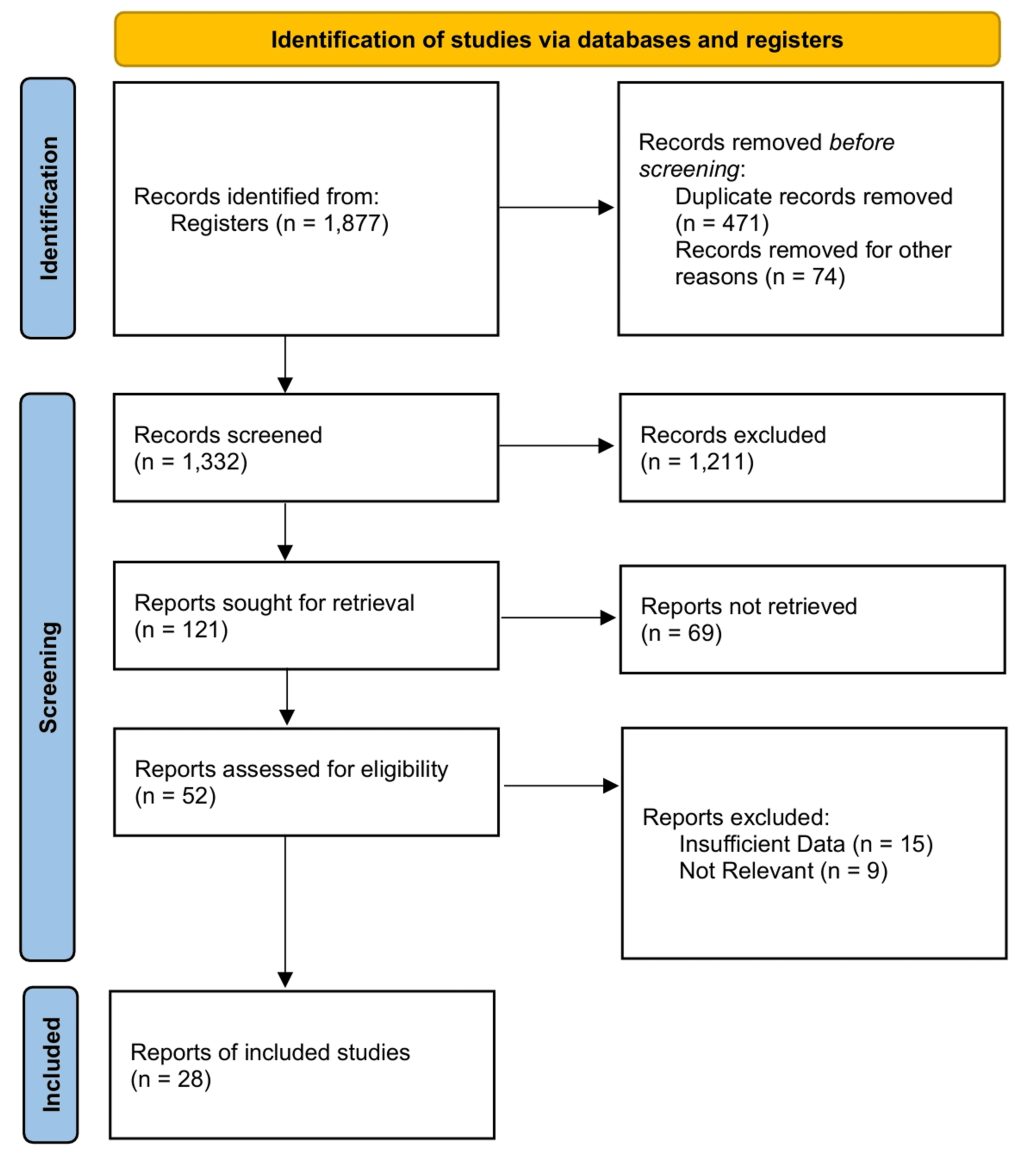
PRISMA flow diagram PRISMA: Preferred Reporting Items for Systematic Reviews and Meta-Analyses

The literature search and study selection processes are detailed in the PRISMA flow diagram (Figure 1). A total of 1,877 records were identified from five electronic databases. After the removal of 471 duplicate records and 74 records for other reasons, 1,332 unique records underwent title and abstract screenings. Of these, 121 reports were retrieved, and 52 full-text articles were assessed for eligibility. Twenty-four studies were excluded because of insufficient outcome data (n=15) or lack of clinical relevance (n=9). Twenty-eight comparative studies met the eligibility criteria and were included in the systematic review and NMA.

Table 1 presents the characteristics of the included studies. IRR for study inclusion demonstrated substantial agreement between the independent reviewers (Cohen's κ=0.63).

**Table 1:** Characteristics of the eligible studies * represents propensity score-matched cohorts for consistency in baseline comparative data. RCT: randomized controlled trial; NIV: non-invasive ventilation; COT: conventional oxygen therapy; HFNC: high-flow nasal cannula; COPD: chronic obstructive pulmonary disease; ICU: intensive care unit; ARF: acute respiratory failure; MV: mechanical ventilation; US: ultrasound; MIMIC-IV: Medical Information Mart for Intensive Care IV

Study (author, year)	Country	Study design	Population/setting	N	Interventions (n)	Comparators (n)
Vargas et al. 2017 [27]	France	Multicenter RCT	Chronic respiratory disorders	143	NIV (71)	COT (72)
Hernández et al. 2022 [28]	Spain	Multicenter RCT	Very high risk of extubation failure	182	NIV (92)	HFNC (90)
Hernández et al. 2016 [29]	Spain	Multicenter RCT	High risk of extubation failure	604	HFNC (290)	NIV (314)
Hernández et al. 2016 [30]	Spain	Multicenter RCT	Low risk of extubation failure	527	HFNC (264)	COT (263)
Arafa 2024 [31]	Saudi Arabia	Single-center RCT	Type II respiratory failure	60	NIV (30)	COT (30)
Rouby et al. 2024 [32]	France/China	Multicenter RCT	High risk, non-COPD	227	NIV+HFNC (128)	COT (99)
Fernandez et al. 2017 [33]	Spain	Multicenter RCT	High risk, non-hypercapnic	155	HFNC (78)	COT (77)
Chen et al. 2023 [34]	China	Retrospective cohort	ICU extubated patients	147	HFNC (75)	COT (72)
Ornico et al. 2013 [35]	Brazil	Single-center RCT	ARF >3 days mechanical vent	38	NIV (20)	COT (18)
Thille et al. 2019 [36]	France	Multicenter RCT	High risk of extubation failure	641	NIV+HFNC (339)	HFNC (302)
Elghonemy et al. 2024 [37]	Egypt	Single-center RCT	Weaned from invasive MV	60	NIV (20); HFNC (20)	COT (20)
Tongyoo et al. 2021 [38]	Thailand	Single-center RCT	Sepsis	222	HFNC (112)	NIV (110)
Thanthitaweewat et al. 2018 [39]	Thailand	Single-center RCT	Medical ICU	58	NIV (29)	COT (29)
Xu et al. 2021 [40]	China	Single-center RCT	High/low risk by US score	98	NIV+HFNC (49)	COT (49)
Munarriz-Ticona et al. 2023 [41]	Peru	Retrospective cohort	COVID-19	206	HFNC (102)	NIV (104)
Xuan et al. 2021 [42]	China	Retrospective Cohort	Sepsis	283	HFNC (167)	NIV (116)
Thille et al. 2021 [43]	France	Post-hoc RCT	COPD subgroup	150	NIV+HFNC (86)	HFNC (64)
Ketan et al. 2023 [44]	India	Single-center RCT	COPD hypercapnic	62	HFNC (30)	NIV (32)
Ge et al. 2023 [45]	China/USA	Retrospective cohort	Obese patients (MIMIC-IV)	308*	HFNC (154)	NIV (154)
Duan et al. 2016 [46]	China	Prospective cohort	Various cough strengths	356	NIV (179)	COT (177)
Casey et al. 2021 [47]	USA	Cluster-crossover RCT	Broad ICU population	751	Protocolized/HFNC (359)	Usual care/COT (392)
Sun et al. 2022 [48]	China	Retrospective cohort	General ICU patients	77	HFNC (40)	COT (37)
Cho et al. 2020 [49]	Korea	Single-center RCT	High risk of reintubation	60	HFNC (31)	COT (29)
Om et al. 2019 [50]	Korea	Retrospective cohort	Acute cardiogenic pulmonary edema	76*	HFNC (38)	COT (38)
Chang et al. 2020 [51]	Taiwan	Retrospective cohort	Heart failure	104	HFNC (58)	NIV (46)
Magdy and Metwally 2023 [52]	Egypt	Single-center RCT	High-risk COPD	230	HFNC (120)	NIV (110)
Shaalan et al. 2024 [53]	Egypt	Single-center RCT	Critically ill	146	HFNC (73)	COT (73)
Fakher 2021 [54]	Egypt	Single-center RCT	Medical ICU	50	NIV (25)	COT (25)

Study Characteristics and Risk of Bias

The 28 included studies comprised 22 RCTs and six non-randomized observational cohort studies that evaluated four distinct post-extubation respiratory support strategies: COT, HFNC, NIV, and the sequential combination of NIV and HFNC (NIV+HFNC).

**Figure 2:**
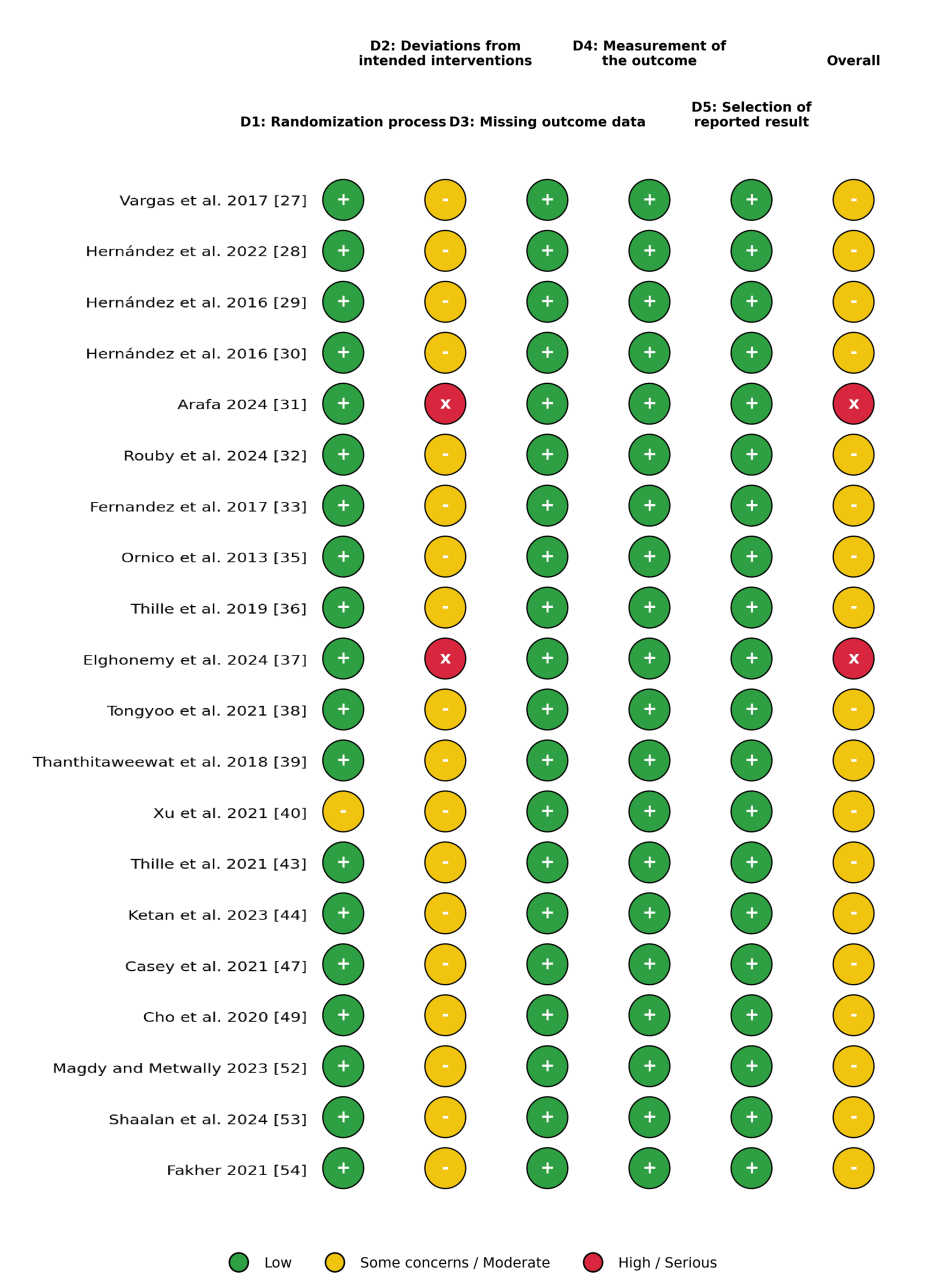
Summary of Cochrane Risk of Bias 2 (RoB 2) judgments for randomized controlled trials

Among the 22 RCTs assessed using the RoB 2 tool, most were adjudicated as having some concerns, driven by deviations from intended interventions that were likely to impact the outcome. Two RCTs were classified as high risk due to deviations from the intended interventions (Figure 2).

**Figure 3:**
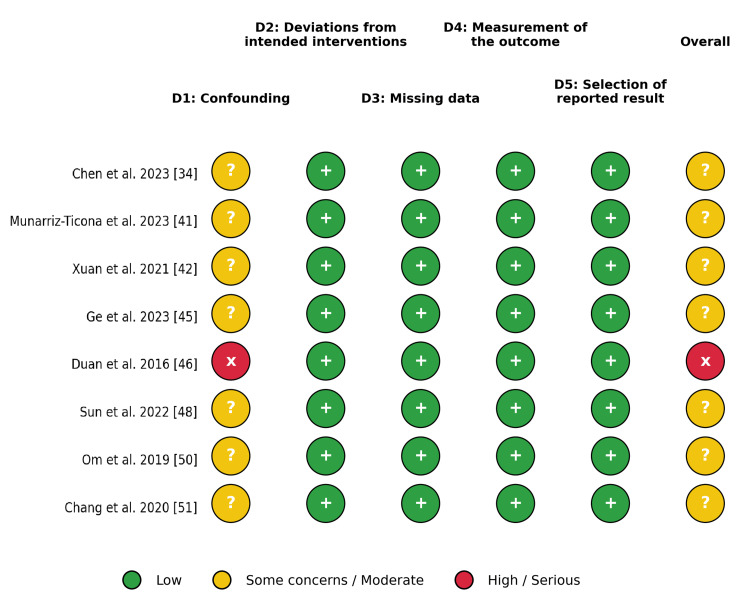
Summary of Risk of Bias in Non-randomized Studies - of Interventions (ROBINS-I) judgments for observational studies

Among the six observational studies assessed using the ROBINS-I tool, five were graded as having a moderate risk of bias, and one was classified as having a serious risk of bias due to critical confounding domains (Figure 3).

**Figure 4:**
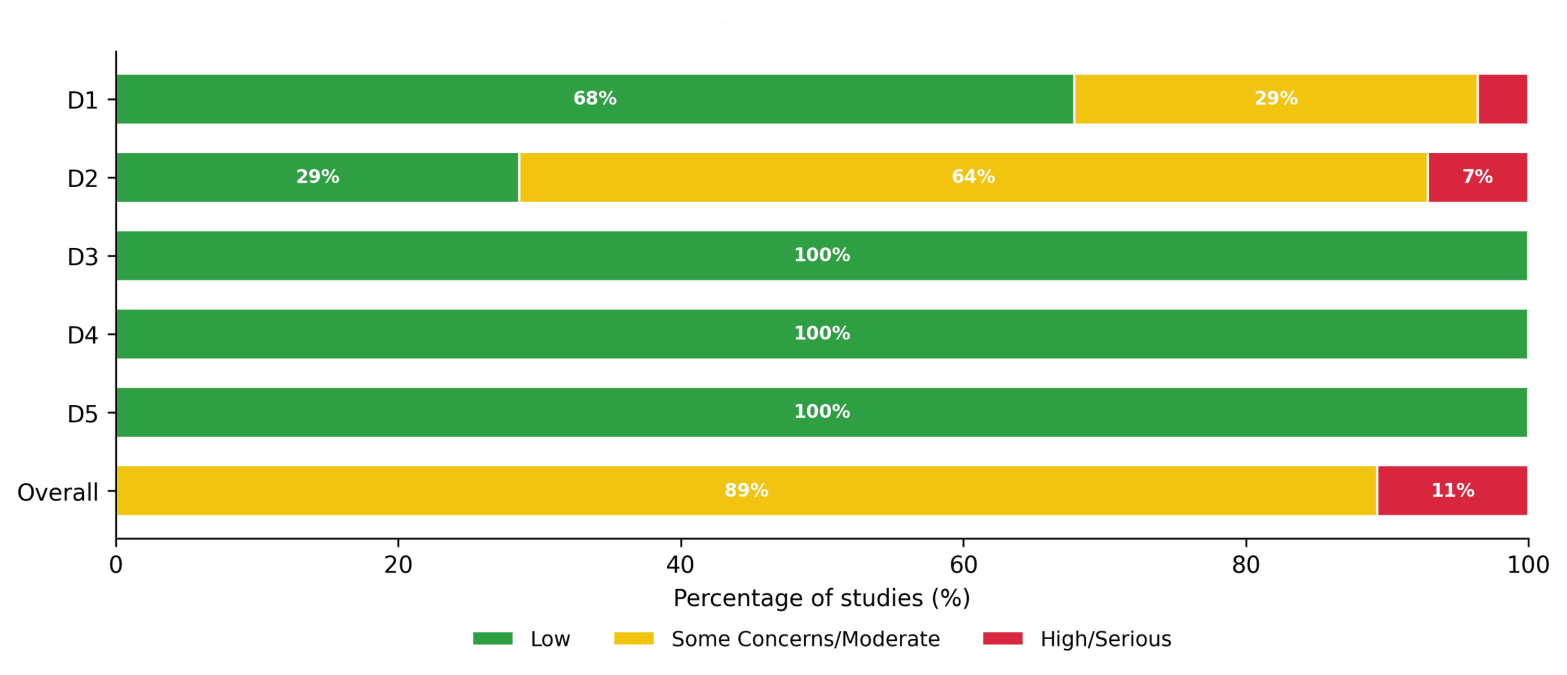
Risk of bias summary across domains

The methodological quality assessments of the included studies are summarized in Figure 4.

Network Geometry and Primary Outcome: Reintubation

**Figure 5:**
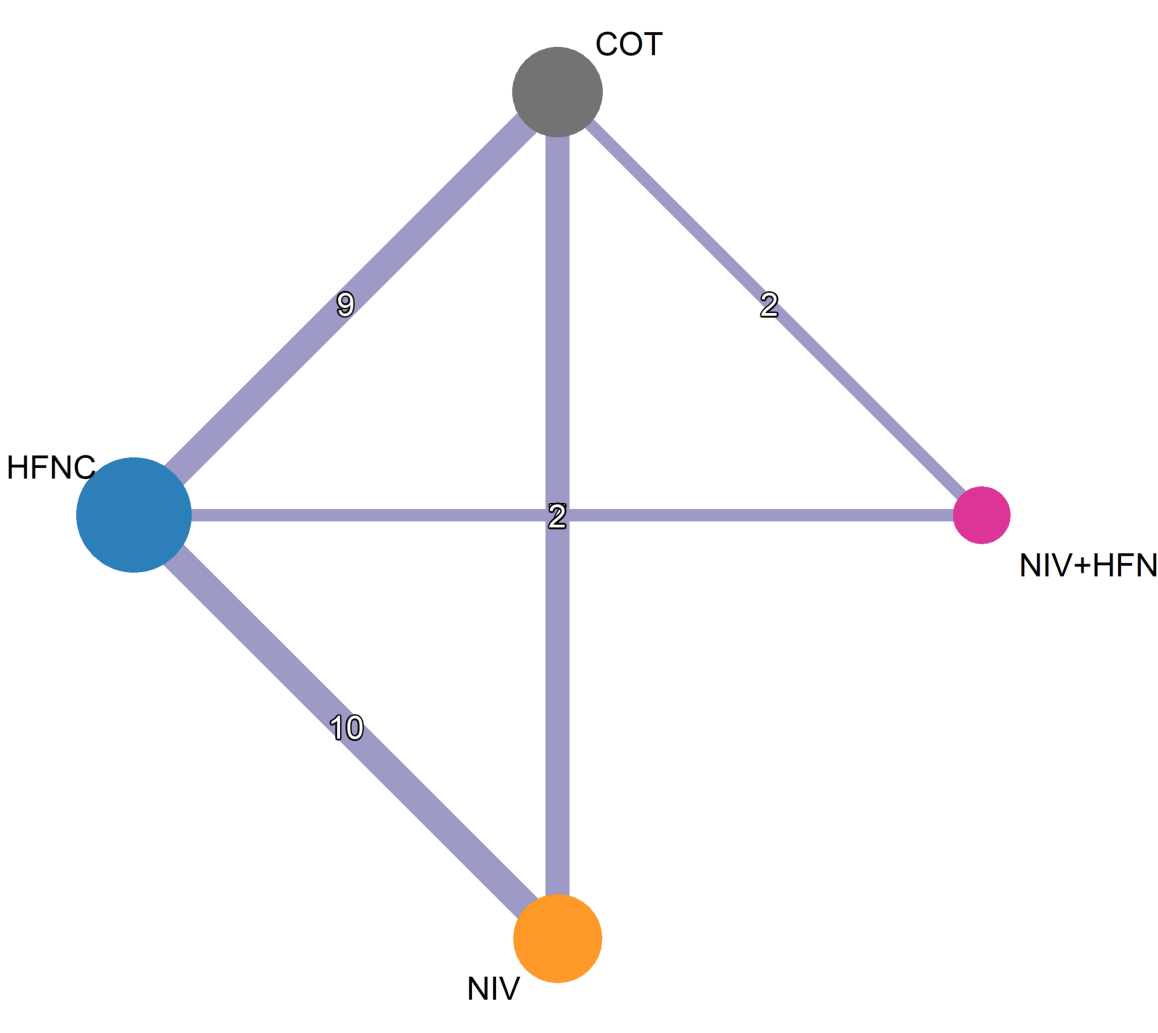
Network geometry plot for post-extubation reintubation Network plot of the 28 included studies. Nodes represent the competing respiratory support strategies. The size of each node is proportional to the total number of patients randomly assigned or matched to that treatment. The thickness of the connecting lines (edges) is proportional to the number of direct comparisons between the connected modalities (number displayed on the edge). COT: conventional oxygen therapy; HFNC: high-flow nasal cannula; NIV: non-invasive ventilation

The network geometry for the primary outcome (reintubation within 48-72 hours) was well-connected, comprising 30 pairwise comparisons across 28 studies (Figure 5). Direct evidence was available for all major nodes, with the highest volume of direct comparisons between HFNC and NIV (10 trials), HFNC and COT (nine trials), and NIV and COT (seven trials).

**Figure 6:**
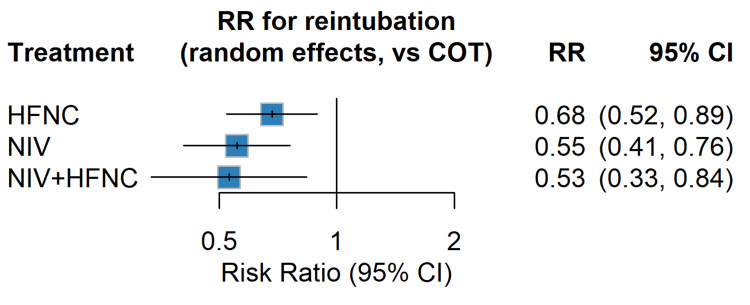
Forest plot of network meta-analysis estimates vs. COT Forest plot illustrating the network meta-analysis estimates for the primary outcome (reintubation within 48-72 hours) of all active respiratory support modalities compared against COT as the common reference. Data are presented as RR with 95% CI. An RR of <1 indicates a lower risk of reintubation. COT: conventional oxygen therapy; HFNC: high-flow nasal cannula; NIV: non-invasive ventilation; RR: risk ratio; CI: confidence interval

In the frequentist random-effects NMA, all three active non-invasive respiratory support strategies significantly reduced the risk of reintubation compared to COT (Figure 6). The greatest reduction in reintubation risk was observed with the combined NIV+HFNC strategy (RR: 0.53; 95% CI: 0.33-0.84), followed closely by NIV alone (RR: 0.55; 95% CI: 0.41-0.76) and HFNC alone (RR: 0.68; 95% CI: 0.52-0.89).

When comparing the active interventions against each other, the network estimates favored NIV+HFNC and NIV over HFNC alone, although these differences did not reach statistical significance (NIV+HFNC vs. HFNC: RR: 0.78; 95% CI: 0.50-1.22; NIV vs. HFNC: RR: 0.81; 95% CI: 0.63-1.05). There was no significant difference between the combined NIV+HFNC strategy and NIV alone (RR: 0.95; 95% CI: 0.58-1.58) (Table 2).

**Table 2:** League table of network meta-analysis estimates for reintubation Lower triangle presents the network meta-analysis estimates (column vs. row). Upper triangle presents direct pairwise estimates (row vs. column). Data are presented as RR with 95% CI. An RR of <1 favors the column treatment (for network estimates) or the row treatment (for direct estimates). COT: conventional oxygen therapy; HFNC: high-flow nasal cannula; NIV: non-invasive ventilation; RR: risk ratio; CI: confidence interval

	NIV+HFNC	NIV	HFNC	COT
NIV+HFNC	NIV+HFNC	-	0.58 (0.33 to 1.03)	0.78 (0.40 to 1.51)
NIV	0.95 (0.58 to 1.58)	NIV	0.91 (0.68 to 1.22)	0.42 (0.27 to 0.66)
HFNC	0.78 (0.50 to 1.22)	0.81 (0.63 to 1.05)	HFNC	0.73 (0.53 to 1.00)
COT	0.53 (0.33 to 0.84)	0.55 (0.41 to 0.76)	0.68 (0.52 to 0.89)	COT

Treatment Ranking

**Figure 7:**
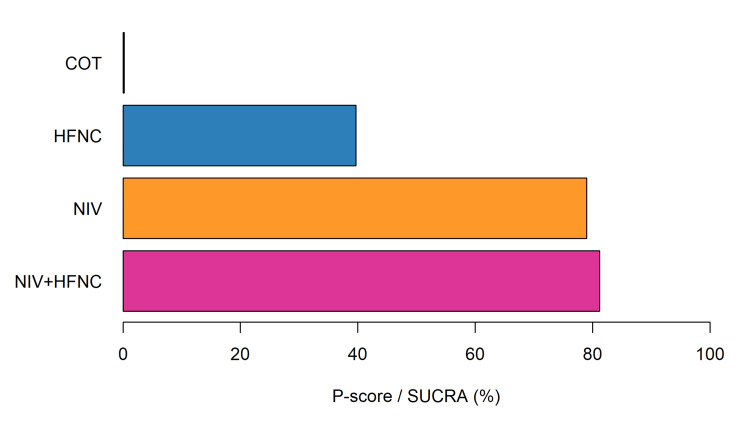
Treatment ranking (P-scores/SUCRA) Bar charts representing the probability rankings (P-scores) of each respiratory support modality for preventing reintubation. Higher scores indicate a greater probability of being the most effective treatment. NIV+HFNC ranked highest (0.8116), followed by NIV (0.7896), HFNC (0.3968), and COT (0.0020). COT: conventional oxygen therapy; HFNC: high-flow nasal cannula; NIV: non-invasive ventilation; SUCRA: surface under the cumulative ranking curve

Treatment hierarchy was established using P-scores (SUCRA equivalents). The combination of NIV+HFNC had the highest probability of being the most effective intervention for preventing reintubation (P-score=0.8116). NIV alone ranked as the second most effective strategy (P-score=0.7896), followed by HFNC alone (P-score=0.3968). COT ranked as the least effective strategy (P-score=0.0020) (Figure 7). However, this ranking should be interpreted alongside effect estimates, confidence intervals, network consistency, and the certainty of evidence, as differences between the active interventions did not reach statistical significance.

Heterogeneity, Inconsistency, and Meta-Regression

**Figure 8:**
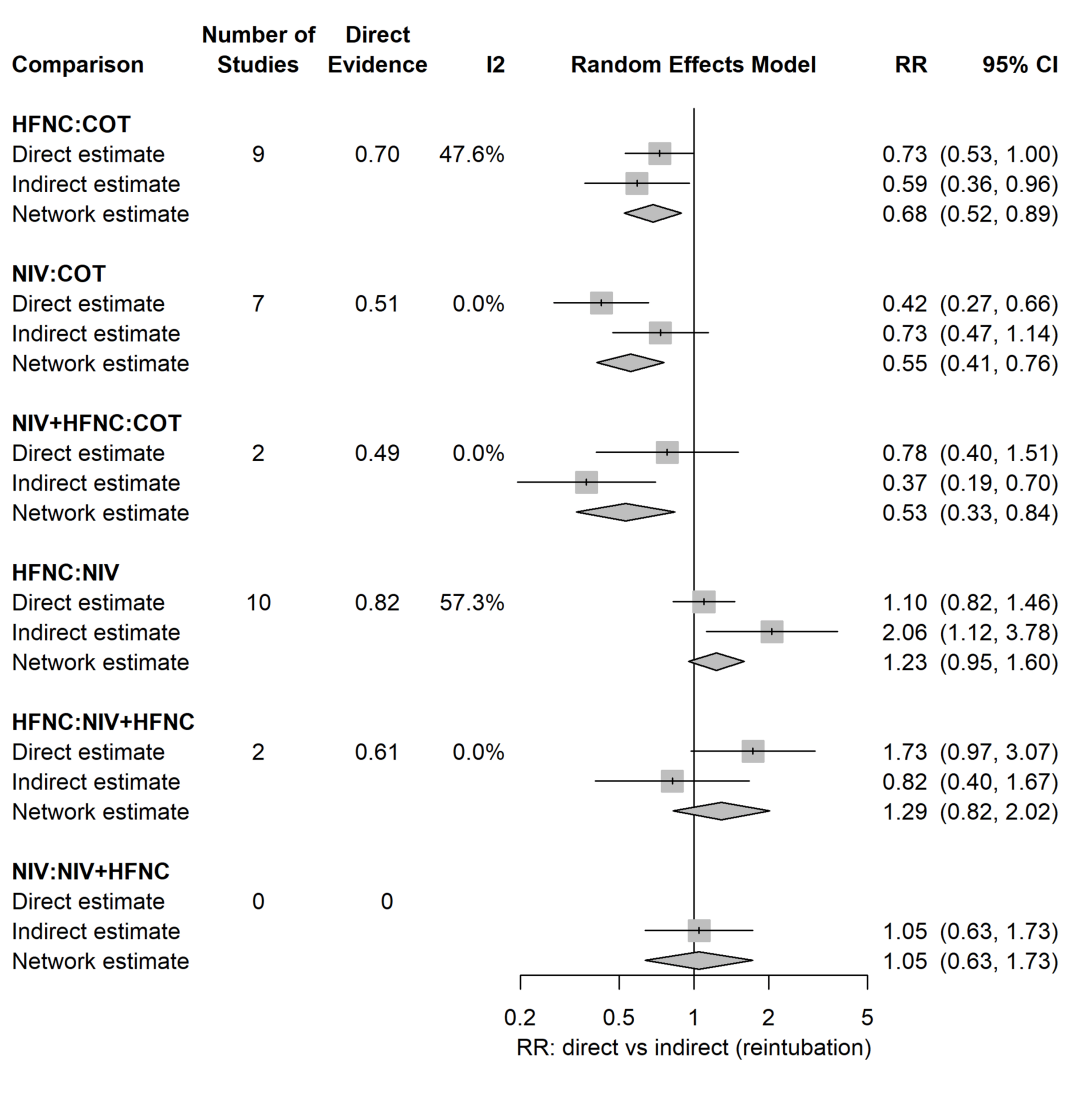
Node-splitting analysis for local inconsistency Forest plot comparing the direct and indirect estimates for each closed loop in the network to evaluate local inconsistency. The analysis demonstrates no statistically significant discrepancies between direct and indirect evidence (all p>0.05), supporting the transitivity of the network. COT: conventional oxygen therapy; HFNC: high-flow nasal cannula; NIV: non-invasive ventilation; RR: risk ratio; CI: confidence interval

Moderate statistical heterogeneity was observed across the network (τ^2^=0.1028; I^2^=48.5% (95% CI: 19.5%-67.1%)). Global inconsistency testing signalled marginal between-design inconsistency (between-designs Q=9.76; df=4; p=0.0447), which was driven by the COT versus HFNC design contrast. However, local inconsistency evaluation using the node-splitting method revealed no statistically significant discrepancies between the direct and indirect estimates for any network loop (all p>0.05) (Figure 8). Nevertheless, the significant global between-design inconsistency indicates potential incoherence within the network, warranting cautious interpretation and suggesting the presence of unmeasured effect modifiers.

To explore the potential sources of heterogeneity, a contrast-level random-effects meta-regression was performed using study design (RCT vs. observational) as a moderator. The study design did not significantly modify the treatment effect (estimate: 0.2812; SE: 0.2173; p=0.196); however, the absence of a statistically significant interaction does not completely rule out residual confounding or bias introduced by observational studies. In addition, a leave-one-out sensitivity analysis demonstrated that the superiority of HFNC over COT remained highly robust; the pooled RR consistently stayed within a tight bound (0.628 to 0.725) across 28 iterative omissions, with the upper confidence limit remaining <1.0 throughout.

Publication Bias and Certainty of Evidence

**Figure 9:**
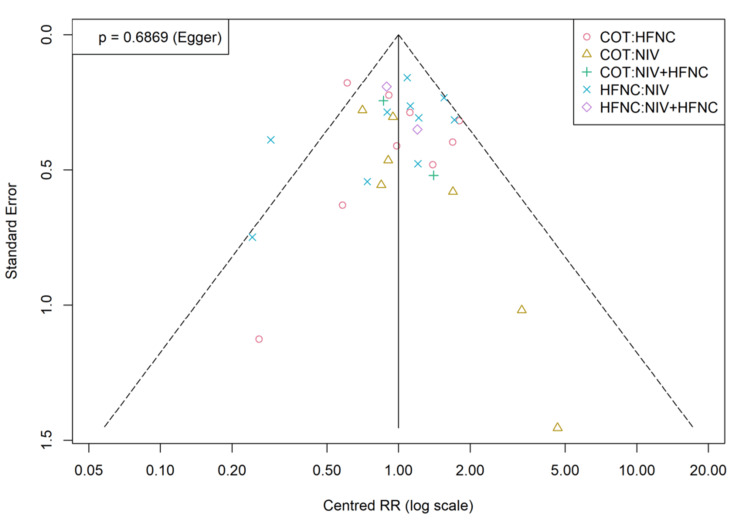
Comparison-adjusted funnel plot Comparison-adjusted funnel plot to assess publication bias and small-study effects. The symmetrical distribution of the plotted points around the vertical axis of zero effect (Egger's test p=0.687) indicates an absence of significant publication bias. COT: conventional oxygen therapy; HFNC: high-flow nasal cannula; NIV: non-invasive ventilation; RR: risk ratio

Visual inspection of the comparison-adjusted funnel plot revealed a symmetrical distribution of effect sizes around the pooled estimates (Figure 9). Quantitative assessment via Egger's linear regression test did not detect statistically significant funnel plot asymmetry (t=0.41; df=28; p=0.687); however, publication bias cannot be definitively excluded based on a non-significant test alone.

**Figure 10:**
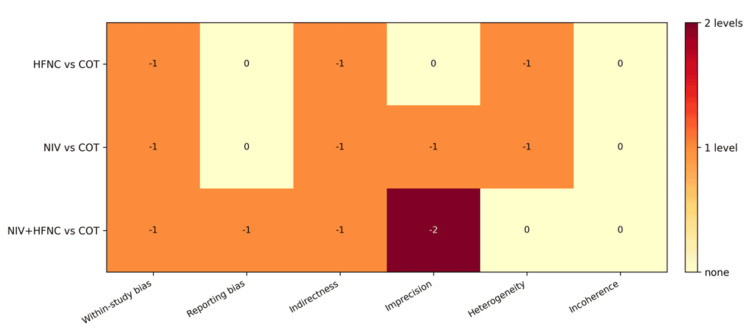
Certainty of evidence (CINeMA framework) Heatmap representation of the CINeMA evaluation for the primary outcome (reintubation). The overall certainty of evidence is graded as moderate for HFNC vs. COT and low for both NIV vs. COT and NIV+HFNC vs. COT, based on evaluations of within-study bias, reporting bias, indirectness, imprecision, heterogeneity, and incoherence. COT: conventional oxygen therapy; HFNC: high-flow nasal cannula; NIV: non-invasive ventilation; CINeMA: Confidence in Network Meta-Analysis

The certainty of the evidence for the primary outcome was evaluated using the CINeMA framework (Figure 10). The certainty of evidence for HFNC versus COT was rated as moderate and downgraded one level due to within-study bias. The evidence for NIV versus COT was rated as low and downgraded for within-study bias, indirectness, and imprecision. The evidence for NIV+HFNC versus COT was also rated as low, downgraded due to within-study bias, reporting bias, indirectness, and major imprecision stemming from wide confidence intervals.

Discussion

In this systematic review and NMA of 28 studies, we evaluated the comparative effectiveness of four post-extubation respiratory support strategies in critically ill adults. Our findings demonstrate that all active non-invasive modalities, HFNC, NIV, and the sequential combination of NIV and HFNC, are superior to COT in preventing reintubation within 48-72 hours. In addition, treatment ranking probabilities (P-scores) identified the combined application of NIV and HFNC as the most efficacious strategy, followed closely by NIV alone, while COT ranked unequivocally as the least effective approach, underscoring a shift in post-extubation management, arguing against the routine use of COT in favor of proactive, advanced respiratory support.

Our findings consolidate and expand upon the fragmented evidence provided by previous pairwise meta-analyses and individual RCTs. The literature has highlighted the limitations of COT, which fails to meet the peak inspiratory flow demands of dyspneic patients and delivers inadequately conditioned gas, leading to impaired mucociliary clearance and atelectasis [1,4,11]. While HFNC addresses these limitations by providing heated, humidified oxygen at high flow rates and generating a modest flow-dependent PEEP [1,10], its ability to unload respiratory muscles is inferior to that of the dual-level pressure support provided by NIV. Our network estimates reflect this physiological hierarchy: while HFNC alone significantly reduced reintubation compared to COT (RR: 0.68; moderate certainty), consistent with previous landmark trials such as the OPERA and Hernández et al. studies [29,30], both NIV and the NIV+HFNC combination achieved numerically greater risk reductions.

The emergence of the sequential NIV+HFNC strategy as the highest-ranked intervention is particularly noteworthy and is biologically plausible. While NIV provides superior physiological support by mitigating auto-PEEP, preventing alveolar collapse, and reducing the work of breathing [2,7], its clinical effectiveness is compromised by patient intolerance, claustrophobia, and interface-induced pressure ulcers [8,10]. Interruptions in NIV therapy bridged with COT can lead to rapid alveolar derecruitment and rebound hypoxemia. By utilizing HFNC during breaks from NIV, clinicians can maintain a continuous level of modest PEEP, ensure precise oxygen delivery, and maximize patient comfort and secretion clearance [36,43]. Our findings statistically validate the synergistic benefits of this approach, driven by large multicenter RCTs, such as the HIGH-WEAN trial, which demonstrated the superiority of this alternating strategy over HFNC alone [36].

Although our NMA favored NIV and NIV+HFNC over HFNC alone, these direct and indirect comparisons did not reach statistical significance (e.g., NIV vs. HFNC: RR: 0.81 (95% CI: 0.63-1.05)). This lack of definitive statistical superiority among active interventions highlights the need for personalized applications based on individual patient phenotypes. For instance, patients at the highest risk of extubation failure, such as those with hypercapnic respiratory failure, severe COPD, or morbid obesity, are likely to derive the most significant physiological benefits from the pressure support provided by NIV or NIV+HFNC [8,44,45]. For patients with purely hypoxemic respiratory failure or those who exhibit severe intolerance to tight-fitting masks, HFNC alone remains a highly effective and evidence-based alternative to COT.

This study represents the most comprehensive synthesis of post-extubation respiratory support to date, incorporating both direct and indirect evidence from a broad spectrum of interventions. The inclusion of the novel NIV+HFNC strategy provides actionable and contemporary insights for critical care practitioners. Our methodology was robust, as we assessed local and global inconsistencies, finding no significant violations of the transitivity assumption. Furthermore, meta-regression and leave-one-out sensitivity analyses confirmed that our estimates were not skewed by the inclusion of high-quality observational data, demonstrating the stability and robustness of the treatment effect of HFNC versus COT.

Despite these strengths, several limitations must be acknowledged. First, the certainty of evidence for comparisons involving NIV and NIV+HFNC was downgraded to low due to within-study bias (lack of blinding in device trials) and imprecision resulting from wide confidence intervals. Second, there was moderate clinical and statistical heterogeneity across the network (I^2^=48.5%). Included studies exhibited variability in their definitions of high-risk populations, criteria for reintubation, and specific device titration protocols (e.g., flow rates, inspiratory positive airway pressure (IPAP)/expiratory positive airway pressure (EPAP) settings). While our meta-regression models explored study design, we were unable to conduct patient-level covariate adjustments for specific subgroups (e.g., COPD vs. non-COPD) due to the aggregate nature of the data. Furthermore, we synthesized prophylactic post-extubation support and rescue therapy for established respiratory deterioration; because these represent clinically distinct indications with different baseline risks, not conducting a quantitative subgroup analysis separating these populations may introduce transitivity concerns. Additionally, not performing an RCT-only sensitivity analysis means residual bias from observational cohorts cannot be entirely excluded. Finally, our primary outcome was restricted to reintubation within 48-72 hours; the impact of these strategies on longer-term mortality or cost-effectiveness warrants further investigation.

## Conclusions

This NMA demonstrates that the prophylactic application of active non-invasive respiratory support, specifically HFNC, NIV, or a sequential combination of both, significantly prevents reintubation in critically ill adults compared to COT. The alternating use of NIV and HFNC ranked highest probabilistically in this network, combining physiological respiratory unloading with patient comfort and sustained oxygenation during rest periods, though direct superiority over other active interventions remains uncertain. The findings support the consideration of HFNC, NIV, or combined strategies in appropriately selected patients at risk of post-extubation failure, while the choice of support should be individualized according to patient risk profile, clinical context, tolerance, and certainty of available evidence.
